# Evaluation of Loopamp *Leishmania* detection kit for the diagnosis of cutaneous leishmaniasis in Ethiopia

**DOI:** 10.1186/s13071-024-06475-3

**Published:** 2024-10-15

**Authors:** Behailu Taye, Roma Melkamu, Fitsumbrhan Tajebe, Ana Victoria Ibarra-Meneses, Desalegn Adane, Saba Atnafu, Mohammed Adem, Gashaw Adane, Mekibib Kassa, Mezgebu Silamsaw Asres, Johan van Griensven, Saskia van Henten, Myrthe Pareyn

**Affiliations:** 1 Department of Immunology and Molecular Biology, University of Gondar, Gondar, Ethiopia; 2 Department of Medical Laboratory Science, Dilla University, Dilla, Ethiopia; 3 Leishmaniasis Research and Treatment Center, University of Gondar, Gondar, Ethiopia; 4 Département de Pathologie et Microbiologie, Faculté de Médecine Vétérinaire, Université de Montréal, Saint-Hyacinthe, QC Canada; 5 Department of Clinical Sciences, Institute of Tropical Medicine, Antwerp, Belgium

**Keywords:** Cutaneous leishmaniasis, Molecular diagnosis, *Leishmania aethiopica*, LAMP

## Abstract

**Background:**

Cutaneous leishmaniasis (CL) in Ethiopia and some parts of Kenya is predominantly caused by *Leishmania aethiopica*. While skin-slit (SS) microscopy is routinely used for CL diagnosis, more sensitive molecular tests are available. The Loopamp™ *Leishmania* detection kit (Loopamp) is a robust loop-mediated isothermal amplification (LAMP) assay with the potential for implementation in primary healthcare facilities. In this study, we comparatively assessed the diagnostic accuracy of four methods currently used to diagnose CL: Loopamp, kinetoplast DNA (kDNA) PCR, spliced leader RNA (SL-RNA) PCR and SS microscopy.

**Methods:**

A study on 122 stored tape disc samples of suspected CL patients was conducted in Gondar, northwestern Ethiopia. Routine SS microscopy results were obtained from all patients. Total nucleic acids were extracted from the tapes and subjected to PCR testing targeting kDNA and SL-RNA, and Loopamp. Diagnostic accuracy was calculated with SS microscopy as a reference test. The limit of detection (LoD) of Loopamp and kDNA PCR were determined for cultured *L. aethiopica* and *Leishmania donovani*.

**Results:**

Of the 122 patients, 64 (52.5%) were identified as CL cases based on SS microscopy. Although the PCR tests showed a sensitivity of 95.3% (95% confidence interval [CI] 91.6–99.1), Loopamp only had 48.4% (95% CI 39.6–57.3) sensitivity and 87.9% (95% CI 82.1–93.7) specificity. The LoD of Loopamp for *L. donovani* was 100-fold lower (20 fg/µl) than that for *L. aethiopica* (2 pg/µl).

**Conclusions:**

The Loopamp™ *Leishmania* detection kit is not suitable for the diagnosis of CL in Ethiopia, presumably due to a primer mismatch with the *L. aethiopica* 18S rRNA target. Further research is needed to develop a simple and sensitive point-of-care test that allows the decentralization of CL diagnosis in Ethiopia.

**Graphical Abstract:**

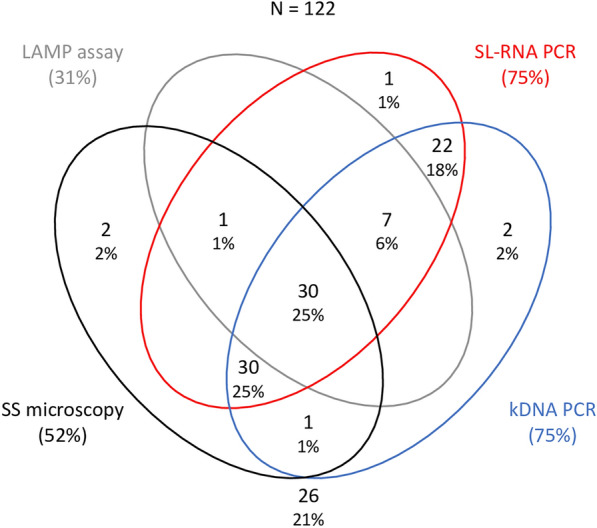

**Supplementary Information:**

The online version contains supplementary material available at 10.1186/s13071-024-06475-3.

## Background

Cutaneous leishmaniasis (CL) is one of the most common neglected tropical diseases (NTDs), manifesting as disfiguring skin lesions. The estimated global CL incidence is 0.7–1.2 million cases annually, with Ethiopia as one of the 10 topmost countries affected [1, 2]. In Ethiopia and parts of Kenya, CL is predominantly caused by *Leishmania aethiopica* [3]. While the annual estimate is 20,000–50,000 new CL cases [2], only 716 were reported to WHO in 2021 from Ethiopia [4]. This indicates severe underreporting of CL in the country, predominantly due to poor surveillance, limited access to healthcare and a lack of good diagnostic tools.

As in most resource-constrained CL endemic countries, CL diagnosis in Ethiopia is performed by microscopic examination of a Giemsa-stained skin-slit (SS) sample [3, 5, 6]. This method has good specificity, but poor sensitivity [7, 8], ranging from 15% to 83% depending on the reference test used [9–11], duration and type of lesion [11], quality of the sample collection, staining and the examiner’s expertise [12]. The patient can experience pain while giving an SS sample [13], in contrast to tape disc samples, which are minimally invasive and simple to collect [14]. Tape disc samples can then be tested for *Leishmania* detection with more sensitive molecular methods, such as PCR [15–17] and loop-mediated isothermal amplification (LAMP) [18].

Minicircle kinetoplast DNA (kDNA) is the most common target for *Leishmania* detection by PCR [19] because of its high copy number [6]. Hence, the kDNA PCR has good diagnostic accuracy and can be combined with invasive [5, 10, 20, 21] and less-invasive [14, 22–24] sample types. The pan-*Leishmania*-specific PCR targeting the mini-exon spliced leader RNA (SL-RNA) has also shown high sensitivity and specificity and has the potential to specifically detect viable parasites [25, 26]. These two PCRs were found to have similar diagnostic performance in a previous study performed on SS samples from Ethiopian CL patients [5]. Although the sensitivity of the PCRs was much better than that of microscopic examination of SS samples when using a composite reference test, these PCRs are not available for the routine diagnosis of CL in Ethiopia, primarily due to the high cost of the reagents and the need for cold-chain storage, sophisticated laboratory facilities and expertise [27].

In contrast, the Loopamp™ *Leishmania* detection kit (Eiken Chemicals, Tokyo, Japan; further referred to as Loopamp) is a robust and simple tool that does not require cold-chain storage. This diagnostic kit detects 18S ribosomal RNA (18S rRNA) and kDNA. It has demonstrated 95.9% sensitivity and 98.8% specificity for visceral (VL) in northwestern Ethiopia on blood samples, and was implemented successfully for VL diagnostics in a rural health center in Sudan [28, 29]. Hence, Loopamp has the potential for enabling the decentralization of CL diagnosis to primary healthcare facilities, which would enable considerably more patients to be diagnosed. Loopamp also showed 84.8–100% sensitivity in studies on CL patients infected with various *Leishmania* species across different endemic areas in the world [18, 27, 30, 31]. Despite Ethiopia being highly endemic for CL, the diagnostic performance of Loopamp for the detection of CL in Ethiopia has never been evaluated. In this study we comparatively assessed the diagnostic performance of the Loopamp™ *Leishmania* detection kit and kDNA and SL-RNA PCRs on non-invasive tape samples of Ethiopian CL patients, with SS microscopy as a reference test.

## Methods

### Study area

The study was conducted at the University of Gondar Comprehensive Specialized Hospital Leishmaniasis Research and Treatment Center (LRTC). This center provides routine care for leishmaniasis patients and has a good clinical laboratory practice compliant-laboratory carrying out immunological and molecular research projects.

### Study samples

For this study, we used stored samples from a previous study that had assessed the diagnostic value of different sample collection tools for CL [32]. This earlier study was conducted on 351 suspected CL patients who had presented at the LRTC between February 2019 and August 2022. Tape disc samples were collected from these patients as previously described [14]. In brief, adhesive plastic tape discs (diameter: 22 mm) were placed on the lesion and the top layer of the stratum corneum stripped off. In the present (sub)study, we used the second tape disc sample collected from patient number 175 to patient number 299 (from the 351 patients in the original study), which had been stored in 300 µl of 1× DNA/RNA Shield reagent (Zymo Research, Irvine, CA, USA) at − 80 °C. The results of the routine microscopic examination of the SS sample (positive/negative and parasite grading as per WHO guidelines [33]) were also recorded.

### Sample size and sampling

Sample size calculation was based on the precision of the sensitivity estimates [34]. Based on an expected sensitivity of 99% for kDNA PCR, 94% for SL-RNA PCR [5] and 95% for Loopamp [18], 5% precision, 5% alpha and an estimated 70% CL prevalence, the minimum sample sizes were 22, 124 and 105 for the three assays, respectively. The largest sample size of 124 was selected for this study. All samples that met the inclusion criteria (written informed consent for secondary use of samples available and samples properly stored) were consecutively used.

### Nucleic acid extraction

Total nucleic acids were extracted from the tape discs in 300 µl of 1× DNA/RNA Shield reagent using the Maxwell LEV Simply RNA Tissue Kit (Promega, Madison, WI, USA) according to the manufacturer’s instructions, but without performing the DNase treatment step, which allowed for the simultaneous isolation of DNA and RNA, as described previously [35]. Samples were processed with the Maxwell 16 automated extractor (Promega) and eluted in 50-µl aliquots. A negative extraction control (NEC, consisting only 1-thioglycerol/homogenization solution and lysis buffer) was included in each batch of 15 samples to check for contamination. To prevent PCR inhibition, extracts were diluted 1:5 in nuclease-free water before undergoing the molecular tests.

### Molecular analysis

Samples were subjected to a probe-based kDNA PCR performed as described previously [5] and to a SYBR-Green SL-RNA PCR, followed by a melting curve analysis, as described previously [25]. For the kDNA PCR, 5 µl of template DNA was used in a 25-µl reaction mix, and for the SL-RNA, 4 µl was used in a 20-µl reaction mix. Samples were run on the QuantStudio 5 Real-Time PCR System (Applied Biosystems, Thermo Fisher Scientific, Waltham, MA, USA). Each run included duplicates of the NECs and non-template controls (nuclease-free water) as checks for contamination, as well as positive PCR controls (*L. donovani* 0.02 ng/µl) to check the PCR performance. PCR results were expressed by cycle threshold (Ct) values. For SL-RNA specifically, a sample was considered positive if the melting temperature (Tm) of the sample was within 0.5 °C of the average of the positive controls in that run.

The Loopamp™ *Leishmania* Detection Kit (Eiken Chemicals) was used in combination with a Genie III® real-time fluorimeter (OptiGene Ltd., Horsham, UK) as described in [27]. Aliquots of template DNA (3 µl each) diluted with 27 µl of nuclease-free water was used in a total reaction volume of 30 µl. The results were expressed as time-to-positivity (Tp) values.

In the case of a sample being negative for CL by all three molecular methods, a PCR that targeted the human beta-globin gene (HBB) with primers and probes (adopted from [36]) was performed as described previously [5] to check for proper sample collection and extraction efficiency. Samples negative for HBB were excluded from the analysis.

The Standards for Reporting Diagnostic Accuracy (STARD) checklist for studies of diagnostic accuracy was followed [37]. The molecular tests were read and checked (blinded to microscopy results) by different researchers.

### Limit of detection of kDNA PCR and Loopamp

To determine the limits of detection (LoD) of the kDNA PCR and Loopamp*,* we isolated DNA from cultured *L. aethiopica* (MHOM/ET/72/L100) and *L. donovani* (MHOM/SD/2007/Ged4) parasites using the QIAamp DNA Blood Kit (Qiagen, Hilden, Germany). A 10-fold serial dilution from 20 pg/µl to 0.02 fg/µl DNA was prepared. All dilutions were performed in five repetitions with the same run.

### Data analysis

Data were entered into EpiData software version 4.6 and transported into Stata version 14.2 (StataCorp LLC, College Station, TX, USA) for analysis. Diagnostic accuracy of the three molecular techniques were evaluated as index tests using SS microscopy as a reference test, with 95% confidence intervals (CI). Correlation between Ct-values of the PCRs and between Tp with Ct-values of both PCRs were determined via Spearman correlation. The association between the SS microscopy-graded parasitic load and Ct/Tp values was checked for each molecular test separately using the Kruskal–Wallis test. The Mann–Whitney U-test was used to test for differences in the Ct-values of kDNA and SL-RNA PCRs between Loopamp-positive and -negative samples. All *P* values < 0.05 were considered to be statistically significant. The LoD was calculated as the lowest dilution providing 95% positive results [38].

## Results

### Sociodemographic and clinical characteristics

From a total of 124 CL suspected patients, two (1.6%) were excluded from the analysis because the results of the HBB PCR were negative; the samples from these two patients were also negative for CL by microscopy. The socio-demographic and clinical characteristics of the remaining 122 suspected CL patients were similar to those previously described [6]. Briefly, 74 (60.7%) samples originated from male patients, and the median age of the patients was 22 (interquartile range [IQR] 18–40) years. The median duration of lesions was 8 (IQR 5–12) months, and the majority (61.5%) of lesions were classified as lupoid cutaneous (LCL), followed by 32.8% mucocutaneous leishmaniasis (MCL) and 5.7% diffuse cutaneous leishmaniasis (DCL) (Additional file 1: Table S1).

### Performance of three molecular methods for CL diagnosis

**Fig. 1:**
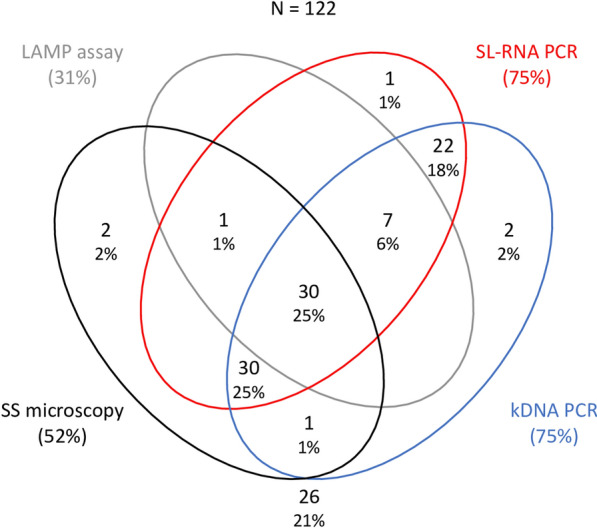
Venn diagram showing the number and proportion of positive results per test. A total of 122 samples were included in this analysis (and used as the denominator to calculate the percentages). Different colors denote the different tests performed: black, microscopy; gray, Loopamp; red, SL-RNA PCR; blue, kDNA. kDNA, Kinetoplast DNA; LAMP, loop-mediated isothermal amplification; SL-RNA, spliced leader RNA; SS, skin-slit

Using SS microscopy as a reference test, 64 patients (52.5%) were identified as CL cases. Of of 122 samples, 92 (75.4%) were positive for CL with kDNA PCR, 91 (74.6%) were positive by SL-RNA PCR and 38 (31.2%) were positive by the Loopamp (Fig. 1). Thirty samples (24.6%) were positive for CL by all four tests, 60 (49.2%) were positive by both PCRs and microscopy, 37 (30.3%) were positive by both PCRs and Loopamp and 89 (73.0%) were positive by the two PCRs only. Two samples were positive by the SL-RNA PCR, but not by the kDNA PCR, and three samples were positive by the kDNA PCR but not by the SL-RNA PCR; in both cases, only one sample could be confirmed by microscopy. Two samples were positive by microscopy, but not by any other test. Of the 64 samples positive by microscopy, less than half (*n* = 31, 48.4%), could be confirmed by Loopamp. All 38 samples that were positive by Loopamp were confirmed by another test.

Both kDNA and SL-RNA PCRs showed a sensitivity of 95.3% (95% CI 91.6–99.1) with microscopy as the reference test. Specificity was 46.5% (95% CI 37.7–55.4) for the kDNA PCR and 48.3% (95% CI 39.4–57.1) for the SL-RNA PCR. The Loopamp demonstrated 48.4% (95% CI 39.6–57.3) sensitivity and 87.9% (95% CI 82.1–93.7) specificity (Table 1).

**Table 1:** Diagnostic performance of three methods (kDNA PCR, SL-RNA PCR and Loopamp) used in this study to diagnose cutaneous leishmaniasis caused by *Leishmania aethiopica*, using skin-slit microscopy as a reference standard

Method	Cases (*n* = 64)^a^		Non-cases (*n* = 58)^a^		Diagnostic performance			
	Positive	Negative	Positive	Negative	Sensitivity (95% CI)	Specificity (95% CI)	PPV (95% CI)	NPV(95% CI)
kDNA PCR	61	3	31	27	95.3 (91.6–99.1)	46.5 (37.7–55.4)	66.3 (57.9–74.7)	90.0 (84.7–95.3)
SL-RNA PCR	61	3	30	28	95.3 (91.6–99.1)	48.3 (39.4–57.1)	67.0 (58.7–75.4)	90.3 (85.1–95.6)
Loopamp	31	33	7	51	48.4 (39.6–57.3)	87.9 (82.1–93.7)	81.6 (74.7–88.5)	60.7 (52.0–69.4)

*CI* Confidence interval,* kDNA* kinetoplast DNA, *NPV* negative predictive value, *PPV* positive predictive value,* SL-RNA* spliced leader RNA

^a^The skin-slit microscopy method was used to distinguish cases and non-cases

**Fig. 2:**
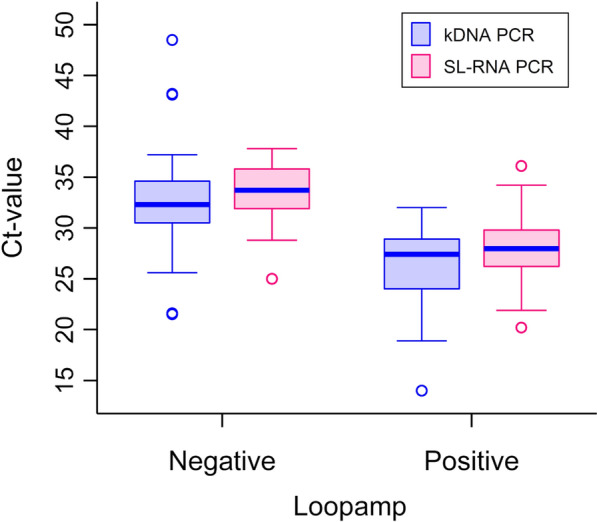
Cycle threshold (Ct) values of kDNA and SL-RNA PCRs for samples that were positive and negative using Loopamp. Boxplots present median values of kDNA (blue) and SL-RNA (pink) PCRs, comparing the values of each test by Loopamp results. The *P*-value for comparing Ct values of kDNA and samples positive and negative by Loopamp was < 0.001. The *P*-value for comparing Ct values of SL-RNA PCR and samples positive and negative by Loopamp was < 0.001. kDNA. kDNA, Kinetoplast DNA; SL-RNA, spliced leader RNA

Samples positive by Loopamp had statistically significant lower (*Z* = 6.34, *P* < 0.001) kDNA Ct-values (median Ct 27.4, IQR 24.0–28.9) compared to the samples that were negative by Loopamp (median Ct 32.3, IQR 30.5–34.6, Fig. 2). Similar results (*Z* = 6.65, *P* < 0.001) were observed for the SL-RNA PCR, where samples positive by Loopamp had significantly lower Ct values (median 27.9, IQR 26.2–29.8) than those negative by Loopamp (median Ct 33.7, IQR 31.9–35.8).

Spearman correlation was strongest at *rs* = 0.749 (*P* < 0.001, *n* = 89) between Ct-values of kDNA and SL-RNA PCRs (Additional file 2: Figure. S1). Correlation of the Loopamp Tp values with Ct values of both PCRs was only *rs* = 0.591 (*P* = 0.0001) (*n* = 37 for kDNA PCR and *n* = 38 for SL-RNA PCR).

Overall, there was a trend between the median Ct-values with SS microscopy grading, which was statistically significant for both PCRs (*H* = 24.0, *df* = 6, *P* < 0.001; Additional file 3: Figure S2). In contrast, there was no significant trend for Loopamp median Tp values with SS microscopy grading (*H* = 6.34, *df* = 6, *P* = 0.384).

### LoD of Loopamp™ *Leishmania* detection kit (Loopamp) and kDNA PCR

While the kDNA PCR showed a LoD of 2 fg/µl (10 fg DNA in the PCR reaction) for *L. aethiopica*, the LoD for Loopamp was only 2 pg/µL (6 pg DNA per reaction; Table 2). The LoD of Loopamp for *L. donovani* was similar to that of the kDNA PCR for *L. aethiopica*, with detection up to 20 fg/µl (60 fg per reaction)*.*

**Table 2:** Loopamp and kinetoplast DNA PCR limit of detection for *Leishmania aethiopica* and Loopamp limit of detection for *Leishmania donovani*

Methods	Species	Tenfold DNA dilutions/µl							
		20 pg	2 pg	200 fg	20 fg	2 fg	0.2 fg	0.02 fg	NTC
kDNA PCR	*Leishmania aethiopica*	Positive	Positive	Positive	Positive	Positive	Negative^a^	Negative	Negative
Loopamp	*L. aethiopica*	Positive	Positive	Negative^a^	Negative	Negative	Negative	Negative	Negative
Loopamp	*Leishmania donovani*	Positive	Positive	Positive	Positive	Negative^a^	Negative	Negative	Negative

* kDNA* Kinetoplast DNA, *NTC* non-template control

^a^One out of replicates that were positive

## Discussion

There is an urgent need for sensitive diagnostic tools for CL that can be employed in primary healthcare facilities [39]. This is especially pressing in settings such as Ethiopia where many cases currently remain undetected due to significant barriers to seeking health care at centralized referral hospitals. Recently, the CL Detect Rapid Test was found to be inferior to microscopy for the diagnosis of CL in Ethiopia [6]. To our knowledge, no other rapid tests are in the pipeline and, therefore, there is an imperative need to evaluate existing tests for their potential to diagnose of CL in Ethiopia.

In our study, the Loopamp *Leishmania* detection kit demonstrated low sensitivity, confirming less than half of the samples positive for CL by SS microscopy. This is well below the 95% sensitivity that was recently specified as a minimum requirement for target product profiles for CL point-of care tests [39]. The Loopamp kit was successfully implemented and optimized at our research site in Ethiopia for the diagnosis of VL, for which it shows good sensitivity, with internal controls that were valid at all times, indicating that there were no technical issues [28]. kDNA and SL-RNA PCRs provided similar results, indicating efficient extraction of both DNA and RNA. The Loopamp LoD was much higher for *L. aethiopica* than for *L. donovani.* As the LoD analysis was performed on *Leishmania* DNA isolated with a Qiagen DNA isolation kit, the poor sensitivity of Loopamp for *L. aethiopica* was once again confirmed*.*

Although *L. aethiopica* was not specified among the species examined during the primer design and kit validation, the kit’s developers show that its primers amplify not only kDNA, but also at least 18S rRNA of CL-causing *Leishmania* species [18]. Despite the lower copy number of 18S rRNA [40], previous studies did show that Loopamp has good sensitivity for a number of CL-causing *Leishmania* species, including *L. tropica*, *L. major* and others, with SS microscopy or internal transcribed spacer 1 (ITS-1) PCR as the reference test [18, 27, 30, 31]. The study of Merdekios et al*.* in Ethiopia also demonstrated very low sensitivity of the ITS-1 rRNA target [5]. Thus, low sensitivity is presumably due to primer mismatch with the variable 18S rRNA gene of *L. aethiopica*. Unfortunately, the manufacturer did not share primer sequences to prove mismatch through in silico analysis. BLAST alignment demonstrates that the 18S sequence of *L. aethiopica* (MHOM/ET/67/L86) varies from that of *L. major* (MHOM/UZ/02/17h) in 26 locations and from *L. tropica* (MHOM/PS/01/ISL590) in six regions (Additional file 4: Figure S3). This could potentially have led to mismatch of the Loopamp primer set to *L. aethiopica* DNA.

Despite the fact that the Loopamp *Leishmania* detection kit appears to be a ready-to-use amplification tool that requires less equipment and time than quantitative PCR [41], it cannot be used for the diagnosis of CL due to *L. aethiopica* because of its limited sensitivity. However, with appropriate primer design, LAMP could still play an important role in the detection of CL in Ethiopia.

We found that the kDNA and SL-RNA PCRs performed equally well for the diagnosis for CL, which is in line with a previous report from Ethiopia [5]. Three samples were missed by both PCR tests, but positive by microscopy; this could have been caused by the different sample types used, with a low level of parasite DNA recovery from the tape samples. Both kDNA and SL-RNA PCRs showed low specificity, probably due to the low sensitivity of the reference test. As such, specificity estimates should be interpreted with care. Emphasizing sensitivity, which is crucial for CL diagnosis, we used the commonly employed SS microscopy method as the reference test to ensure the detection of true CL cases. Despite its high sensitivity, primary healthcare centers cannot use PCR for routine CL diagnosis due to its lack of field adaptability, the high costs and the requirement for cold-chain storage of reagents [27, 41].

There are a number of limitations associated with this study, including the low number of negative samples and the assumption that every CL case was caused by *L. aethiopica*. Recent evidence indicates that a minor proportion of the CL cases in northern Ethiopia are caused by *L. donovani*, although this could also be a hybrid species [42]. Despite the possibility that few study participants had CL that was possibly caused by *L. donovani*, based on our findings we can still conclude that the Loopamp *Leishmania* detection kit is not suitable for the diagnosis of CL in Ethiopia. Lastly, we used non-invasive tape discs to compare molecular techniques in this project. Recent research indicates that tape discs can be contaminated as early as during sample collection, which could potentially lead to false positive results for all three molecular tests employed in this study [32]. Due to this limitation, we cannot draw firm conclusions as to whether patients who were positive for CL by the molecular tests but negative by microscopy are really CL cases, or the results are due to contamination. Also, the specificity of the molecular tests used could be higher than demonstrated here if used with another sample type. Therefore, we decided to focus on whether these molecular techniques are able to detect definite CL cases (which can be identified with microscopy as a reference test) and meet the minimum sensitivity of 95% as specified in a recently published target product profile [39].

## Conclusions

The Loopamp *Leishmania* detection kit (Eiken Chemicals) is not appropriate for the diagnosis of CL due to *L. aethiopica*. Further research is needed to develop simple point-of-care tests with high sensitivity that allow the diagnosis and care of CL patients in Ethiopia to be decentralized.

## Supplementary Information

**Additional file 1: Table S1.** Sociodemographic and clinical characteristics of the study participants. **Table S2.** Diagnostic performance of kDNA PCR, SL-RNA PCR, and LAMP assay for CL diagnosis, using SSM as a reference standard. **Table S3.** User-friendliness and cost per test of SSM, kDNA PCR, SL-RNA PCR, and LAMP assay for CL diagnosis.

**Additional file 2: Figure S1.** Spearman correlation of Ct and Tp values obtained through kDNA and SL-RNA PCRs and the Loopamp.

**Additional file 3: Figure S2.** Boxplot for comparison of Loopamp Tp and Ct-values of kDNA and SL-RNA PCRs with microscopy graded parasite load.

**Additional file 4: Figure S3.** Alignment of the 18S sequence of *L. aethiopica* (GenBank FN677356.1) to L. major (GenBank FN677357.1) and *L. tropica* (GenBank FN677345.1).

**Additional file 5.** Molecular data generated and/or analyzed during the present study.

## Data Availability

Data supporting the conclusions of this article are included within the article and its additional files. Clinical data generated and/or analyzed during the present study are not publicly available, but are available from the corresponding author upon reasonable request.

## Abbreviations

CL: Cutaneous leishmaniasis
Ct: Cycle threshold
kDNA: Kinetoplast DNA
LAMP: Loop-mediated isothermal amplification
LoD: Limit of detection
rRNA: Ribosomal RNA
SL-RNA: Spliced leader RNA
SS: Skin-slit
Tp: Time-to-positivity
