# Supplementary material for: Evaluation of Loopamp Leishmania detection kit for the diagnosis of cutaneous leishmaniasis in Ethiopia
Source: Parasit Vectors. 2024 Oct 15;17:431. doi: 10.1186/s13071-024-06475-3 (PMC11481786; doi:10.1186/s13071-024-06475-3)
Supplement: Supplementary file 1 — Additional file 1: Table S1. Sociodemographic and clinical characteristics of the study participants. Table S2. Diagnostic performance of kDNA PCR, SL-RNA PCR, and LAMP assay for CL diagnosis, using SSM as a reference standard. Table S3. User-friendliness and cost per test of SSM, kDNA PCR, SL-RNA PCR, and LAMP assay for CL diagnosis. [file 13071_2024_6475_MOESM1_ESM.docx]

Additional file 1: Table S1: Sociodemographic and clinical characteristics of the study participants

| Characteristic | Total N = 122 |
| --- | --- |
| Sex, n (%) |  |
| Male | 74 (60.7) |
| Female | 48 (39.3) |
| Age (years), median (IQR) | 22.5 (18-40) |
| Duration of lesion (months), median (IQR) | 8 (5-12) |
| CL type, n (%) |  |
| LCL | 75 (61.5) |
| MCL | 40 (32.8) |
| DCL | 7 (5.7) |

*Note: N: total number of patients, IQR: interquartile range, n: number of patients for variable, %: percent, LCL: localized cutaneous leishmaniasis, MCL: muco-cutaneous leishmaniasis, and DCL: diffuse cutaneous leishmaniasis*

Table S2: Diagnostic performance of kDNA PCR, SL-RNA PCR, and LAMP assay for CL diagnosis, using SSM as a reference standard

| Method | Case, n=64 | | Non-case, n=58 | | Diagnostic performance | | | |
| --- | --- | --- | --- | --- | --- | --- | --- | --- |
|  | Positive | Negative | Positive | Negative | Sensitivity (95% CI) | Specificity (95% CI) | PPV (95% CI) | NPV(95% CI) |
| kDNA PCR | 61 | 3 | 31 | 27 | 95.3 (91.6-99.1) | 46.5 (37.7-55.4) | 66.3 (57.9-74.7) | 90.0 (84.7-95.3) |
| SL-RNA PCR | 61 | 3 | 30 | 28 | 95.3 (91.6-99.1) | 48.3 (39.4-57.1) | 67.0 (58.7-75.4) | 90.3 (85.1-95.6) |
| LAMP assay | 31 | 33 | 7 | 51 | 48.4 (39.6-57.3) | 87.9 (82.1-93.7) | 81.6 (74.7-88.5) | 60.7 (52.0-69.4) |

*Note: kDNA kinetoplast deoxyribonucleic acid, PCR: polymerase chain reaction, SL-RNA: spliced leader ribonucleic acid, LAMP: loop-mediated isothermal amplification, PPV: positive predictive value, NPV: negative predictive value, and CI: confidence interval*

Table S3: User-friendliness and cost per test of SSM, kDNA PCR, SL-RNA PCR, and LAMP assay for CL diagnosis

| **Aspects of user-friendliness** | **Laboratory diagnosis methods** | | | |
| --- | --- | --- | --- | --- |
|  | **SSM** | **kDNA PCR** | **SL-RNA PCR** | **LAMP** |
| **Time factor** |  |  |  |  |
| Time to prepare samples | 40 minutes | 151minutes | 169 minutes | 95 minutes |
| Time to analyze | 30 minutes | 139minutes | 140 minutes | 45 minutes |
| Total time | 1:10 hours | 4:50 hours | 5:10 hours | 2:20 hours |
| Stability time of test reagents | 30 months | 20 months | 16 months | 12 months |
| Stability time of quality control | 30 months | 20 months | 16 months | 12 months |
| **Ease of performance,**  **easy/moderate/difficult, %** |  |  |  |  |
| Sample preparation | 100/0/0 | 0/83.3/16.7 | 0/66.7/33.3 | 100/0/0 |
| Operation of analyzer | 83.3/16.7/0 | 0/83.3/16.7 | 0/66.7/33.3 | 83.3/16.7/0 |
| Interpretation of result | 83.3/16.7/0 | 0/83.3/16.7 | 0/66.7/33.3 | 83.3/16.7/0 |
| **Susceptibility to error in procedure, low/medium/high, %** |  |  |  |  |
| Sample preparation | 66.7/33.3/0 | 0/33.3/66.7 | 0/33.3/66.7 | 50/33.3/16.7 |
| Operation of analyzer | 50/50/0 | 33.3/16.7/50 | 33.3/16.7/50 | 50/50/0 |
| Interpretation of result | 50/50/0 | 33.3/16.7/50 | 33.3/0/66.7 | 50/50/0 |
| **Field applicable and other, yes/no, %** |  |  |  |  |
| **No** need for specific sample storage other than cold chain | 83.3/16.7 | 16.7/83.3 | 16.7/83.3 | 83.3/16.7 |
| Refrigerator **not** required to store reagent and controls | 83.3/16.7 | 16.7/83.3 | 16.7/83.3 | 83.3/16.7 |
| Device portable | 100/0 | 50/50 | 50/50 | 100/0 |
| Device battery operated | 33.3/66.7 | 16.7/83.3 | 16.7/83.3 | 83.3/16.7 |
| **No** requirement of extra instruments | 100/0 | 0/100 | 0/100 | 83.3/16.7 |
| Possibility to quantify | 83.3/16.7 | 100/0 | 100/0 | 66.7/33.3 |
| Potential of high-throughput screening, n | 1 | 87-90 | 87-90 | 5-6 |
| SUS score | 67.5 | 57.5 | 62.5 | 75 |
| Cost per test (ETB) | 0.725 | 866.35 | 456.3 | 754.31 |

*SSM: skin-slit microscopy, kDNA PCR: kinetoplast deoxyribonucleic acid polymerase chain reaction, SL-RNA: spliced leader ribonucleic acid, LAMP: loop-mediated isothermal amplification, SUS: system usability scale, ETB: Ethiopian birr, n: number, and %: percent*
