# Supplementary figures and images for: Evaluation of Loopamp Leishmania detection kit for the diagnosis of cutaneous leishmaniasis in Ethiopia
Source: Parasit Vectors. 2024 Oct 15;17:431. doi: 10.1186/s13071-024-06475-3 (PMC11481786; doi:10.1186/s13071-024-06475-3)

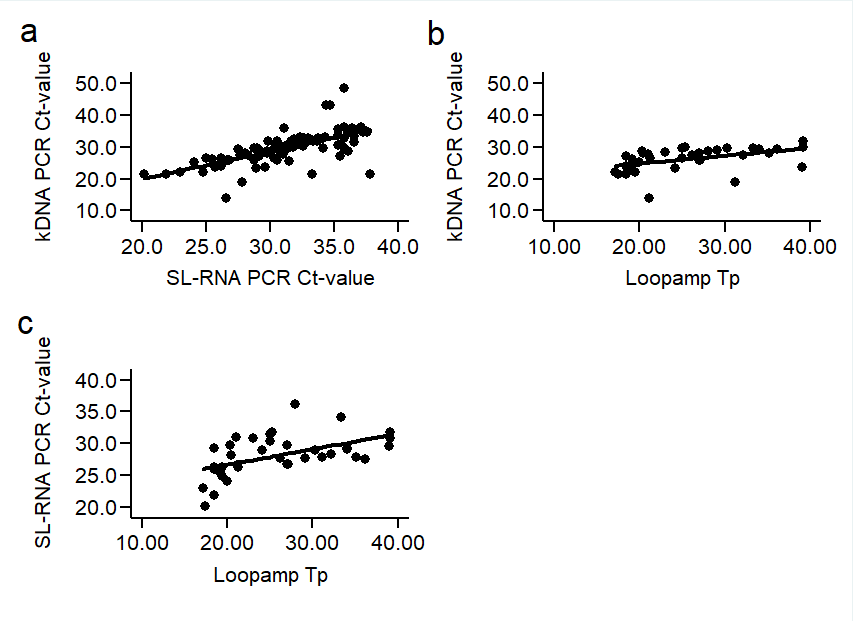

Supplement: Supplementary file 2 — Additional file 2: Figure S1. Spearman correlation of Ct and Tp values obtained through kDNA and SL-RNA PCRs and the Loopamp. [file 13071_2024_6475_MOESM2_ESM.tif]

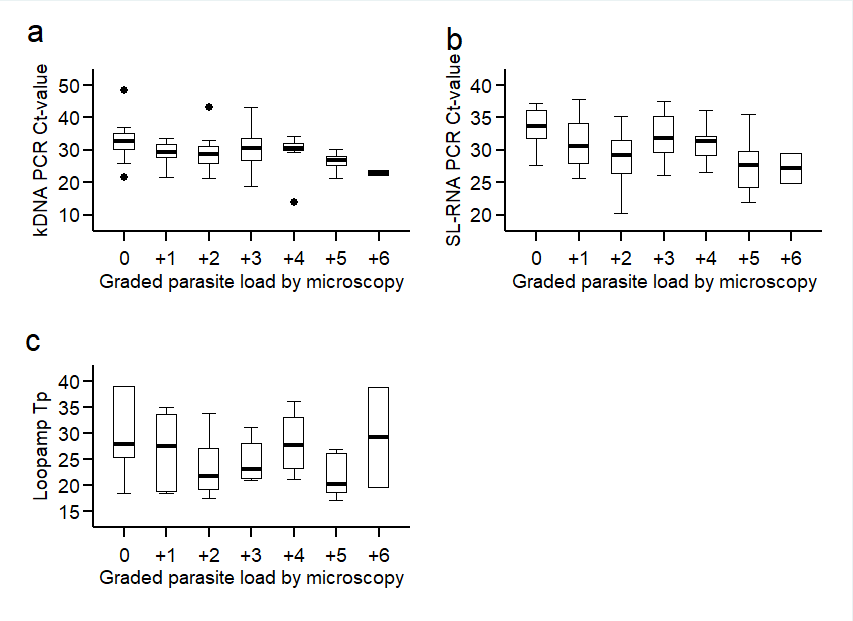

Supplement: Supplementary file 3 — Additional file 3: Figure S2. Boxplot for comparison of Loopamp Tp and Ct-values of kDNA and SL-RNA PCRs with microscopy graded parasite load. [file 13071_2024_6475_MOESM3_ESM.tif]

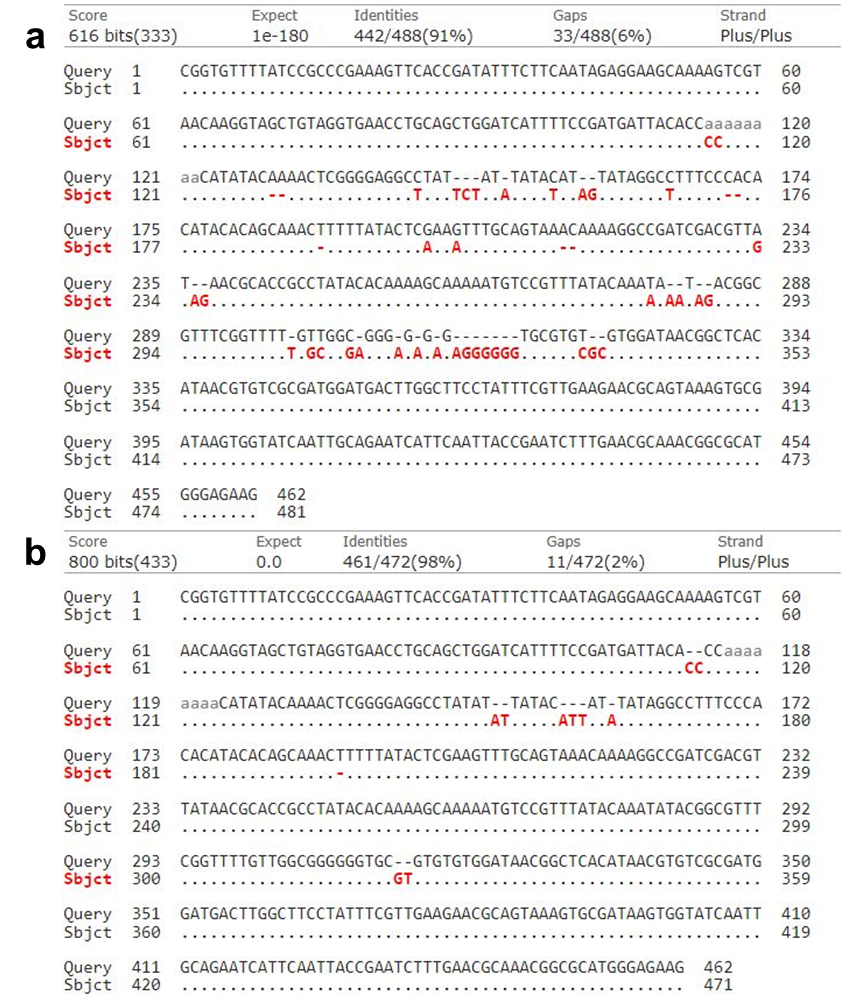

Supplement: Supplementary file 4 — Additional file 4: Figure S3. Alignment of the 18S sequence of L. aethiopica (GenBank FN677356.1) to L. major (GenBank FN677357.1) and L. tropica (GenBank FN677345.1). [file 13071_2024_6475_MOESM4_ESM.tif]
